# Computational prediction of potential inhibitors for SARS-COV-2 main protease based on machine learning, docking, MM-PBSA calculations, and metadynamics

**DOI:** 10.1371/journal.pone.0267471

**Published:** 2022-04-22

**Authors:** Isabela de Souza Gomes, Charles Abreu Santana, Leandro Soriano Marcolino, Leonardo Henrique França de Lima, Raquel Cardoso de Melo-Minardi, Roberto Sousa Dias, Sérgio Oliveira de Paula, Sabrina de Azevedo Silveira

**Affiliations:** 1 Department of Computer Science, Universidade Federal de Viçosa, Viçosa, Minas Gerais, Brazil; 2 Department of Biochemistry and Immunology, Universidade Federal de Minas Gerais, Belo Horizonte, Minas Gerais, Brazil; 3 Department of Computer Science, Universidade Federal de Minas Gerais, Belo Horizonte, Minas Gerais, Brazil; 4 School of Computing and Communications, Lancaster University, Lancaster, United Kingdom; 5 Department of Exact and Biological Sciences, Universidade Federal de São João del-Rei, Sete Lagoas Campus, Sete Lagoas, Minas Gerais, Brazil; 6 Department of General Biology, Universidade Federal de Viçosa, Viçosa, Minas Gerais, Brazil; 7 Department of Microbiology, Universidade Federal de Viçosa, Viçosa, Minas Gerais, Brazil; University of Parma, ITALY

## Abstract

The development of new drugs is a very complex and time-consuming process, and for this reason, researchers have been resorting heavily to drug repurposing techniques as an alternative for the treatment of various diseases. This approach is especially interesting when it comes to emerging diseases with high rates of infection, because the lack of a quickly cure brings many human losses until the mitigation of the epidemic, as is the case of COVID-19. In this work, we combine an in-house developed machine learning strategy with docking, MM-PBSA calculations, and metadynamics to detect potential inhibitors for SARS-COV-2 main protease among FDA approved compounds. To assess the ability of our machine learning strategy to retrieve potential compounds we calculated the Enrichment Factor of compound datasets for three well known protein targets: HIV-1 reverse transcriptase (PDB 4B3P), 5-HT2A serotonin receptor (PDB 6A94), and H1 histamine receptor (PDB 3RZE). The Enrichment Factor for each target was, respectively, 102.5, 12.4, 10.6, which are considered significant values. Regarding the identification of molecules that can potentially inhibit the main protease of SARS-COV-2, compounds output by the machine learning step went through a docking experiment against SARS-COV-2 M^pro^. The best scored poses were the input for MM-PBSA calculations and metadynamics using CHARMM and AMBER force fields to predict the binding energy for each complex. Our work points out six molecules, highlighting the strong interaction obtained for M^pro^-mirabegron complex. Among these six, to the best of our knowledge, ambenonium has not yet been described in the literature as a candidate inhibitor for the SARS-COV-2 main protease in its active pocket.

## Introduction

During an outbreak, it is necessary to quickly respond to the unknown pathogen to avoid the uncontrolled spread of the disease. Just like what happened with the novel COVID-19 disease, caused by the Severe Acute Respiratory Syndrome Coronavirus 2 (SARS-COV-2), that appeared for the first time in Wuhan, China at the end of 2019, spreading quickly all over the globe. In approximately one year, the infection reached more than 170 million cumulative confirmed cases and caused over than 3.5 million deaths (https://covid19.who.int/; as of Jun 2021). Considering that the development of new drugs is expensive and time-consuming, in this scenario computational strategies can potentially speed up the process of drug discovery. The repurposing of existing drugs to treat new diseases can accelerate the approval process, giving a quick response against unknown pathogens [1]. The use of the antiviral Remdesivir is an example, as this drug was initially indicated for the treatment of Ebola, and in October 2020, FDA approved it for use against COVID-19 [2]. However, the mortality rate for patients treated with Remdesivir is still quite high and does not differ significantly from placebo treatment in clinical trials [3, 4]. This shows that treatment with this antiviral alone is still not enough and further research to identify other promising drugs should continue.

Drug development often starts with the identification of key molecules, generally proteins, also called targets, which are crucial for the treatment of a specific disease. High-throughput screening (HTS) experiments are performed aiming to identify compounds that interact with a target protein to achieve a biological purpose, and these compounds can be used as drug candidates. However, designing HTS experiments is expensive, which requires time and resources such as advanced laboratories having chemical and biological libraries [5, 6]. The problem increases when considering the drug development as a whole since the cost of all process is upwards of US$2.8 billion [7]. Moreover, there is a lack of correlation between *in vivo* and *in vitro* assays, since HTS is not able to predict clinical failures, such as side effects and toxicity problems. To address these challenges, computational methods, such as virtual screening (VS), have been developed to mitigate costs and improve productivity in drug development [8–11].

VS is a computational approach in drug discovery, which aims to predict drug-like small molecules that can bind a drug target, generally, a protein [12]. The VS pipeline has the potential to highly reduce the cost and time required for HTS experiments, discarding unlikely compound-target pairs and, as result, potential active combinations are selected [5]. The binding affinity between a protein target and a ligand candidate is assessed by scoring functions associated with the method, in which potential ligands are ranked according to their binding capability. There are two main approaches of VS, structure-based and ligand-based. These can also be combined, generating a hybrid approach [13].

The structure-based virtual screening (SBVS) uses structural information of a protein target, such as biding pockets, to dock a ligand candidate to obtain energy predictions. It is highly dependent on the three-dimensional structure of the protein, which can be considered as a limitation because there is a considerable gap between the availability of protein sequences and protein structures. According to The UniProt Consortium, there are 189 million sequence records in Uniprot, a catalogue of all known protein sequences. On the other hand, in Protein Data Bank (PDB), the catalogue of all known structures, there are 175,000 macromolecules [14]. Therefore, depending on the target, it is necessary to create a protein structure model first, which can potentially impact on the quality of the final result [15, 16]. Furthermore, docking techniques may disregard dynamic features of the protein and, thus, the three-dimensional structure may not represent the most favorable conformation for binding a ligand that would be highly favorable in another thermodynamic state. For these reasons, structure-based methods are more commonly used for optimization, along with some other techniques, such as molecular dynamics [17]. The primary characteristic that distinguishes SBVS tools is how spatial conformations are ranked, according to the score function. There is a current trend to replace classical score functions with more sophisticated methods involving machine learning, to increase the accuracy of the tools [18]. Thus, recent methods look for improvements in the calculation of score functions, such as AtomNet [19], which uses deep convolutional neural network, and SIEVE [20], which uses features of intermolecular binding energy.

Another popular class of methods to perform virtual screening is the ligand-based, which explores chemical and structural features among small molecules to identify compounds having a similar pharmacological activity to the protein. Therefore, it requires a large volume of known data for the specific target, opening space for the employment of a variety of machine learning methods [21]. The main algorithms used for this purpose are support vector machines, k-nearest neighbors, random forests, genetic algorithms, and artificial neural networks, which can be combined [6, 22, 23]. There are some data structures usually employed to represent molecules in ligand-based virtual screening (LBVS) algorithms: graphs, based on the molecular structure [24, 25], fingerprint vectors, which characterize the ligand in terms of its physicochemical and structural properties [23, 26, 27], and ACFs (Atom-Centered Fragments) [28, 29]. We can highlight some examples involving each of the structures. LiSiCA [24] is a software that applies the click algorithm to find and rank similarities between pairs of two or three-dimensional molecules, represented in the shape of graphs. The LBVS [29] is a web server that uses BindingDB [30] and ChEMBL [31] as data sources for the Naive Bayes classifier, whose features are ACFs (Atom-Centered Fragments). Another web server is HybridSim-VS which performs virtual screening ligand prediction based on 2D and 3D fingerprints to calculate the similarity between molecules [27].

SARS-COV-2 is an enveloped RNA positive genome virus with about 30kb that encodes two polyproteins, which are cleaved to constitute the 16 non-structural proteins and four structural proteins, essential to virus cycle [8, 32]. The polyproteins cleaveage is mostly done by the main-protease (M^pro^) which makes this protein a potential drug target to inhibit virus replication [10].

In this work, we combine an in-house developed machine learning strategy, docking, MM-PBSA calculations, and metadynamics to identify molecules approved by the Food and Drug Administration (FDA) that can potentially act as inhibitors of the enzyme activity of the main protease of SARS-COV-2, which is relevant for interrupting the viral cycle. Our computational strategy is hybrid as it combines LBVS and SBVS: (i) We devised a machine learning strategy that couples different molecule fingerprints to perform a first step of LBVS. (ii) Next, the resulting molecules went through SBVS steps. These molecules are docked against the target protein (SARS-COV-2 M^pro^) using Autodock Vina. The poses with lowest vina scores show the preliminary favorable fit of the ligand inside the M^pro^ active site. Finally, we selected the best-scored poses to perform Molecular Mechanics Poisson-Boltzmann Surface Area (MM-PBSA) and metadynamic simulations using two distinct force fields, CHARMM and AMBER. These simulations intended to predict the binding energy for each complex and inspect the impact of the force field on this kind of simulation.

This work aims to identify, through computational techniques, promising molecules to interact with the target protein. The results obtained here serve as input for subsequent in vitro assays to validate the inhibition potential suggested by in silico experiments. All the source code and input datasets are available at https://github.com/IsabelaGomes/Prediction_SARSCOV2_inhibitors.

## Materials and methods

**Fig 1:**
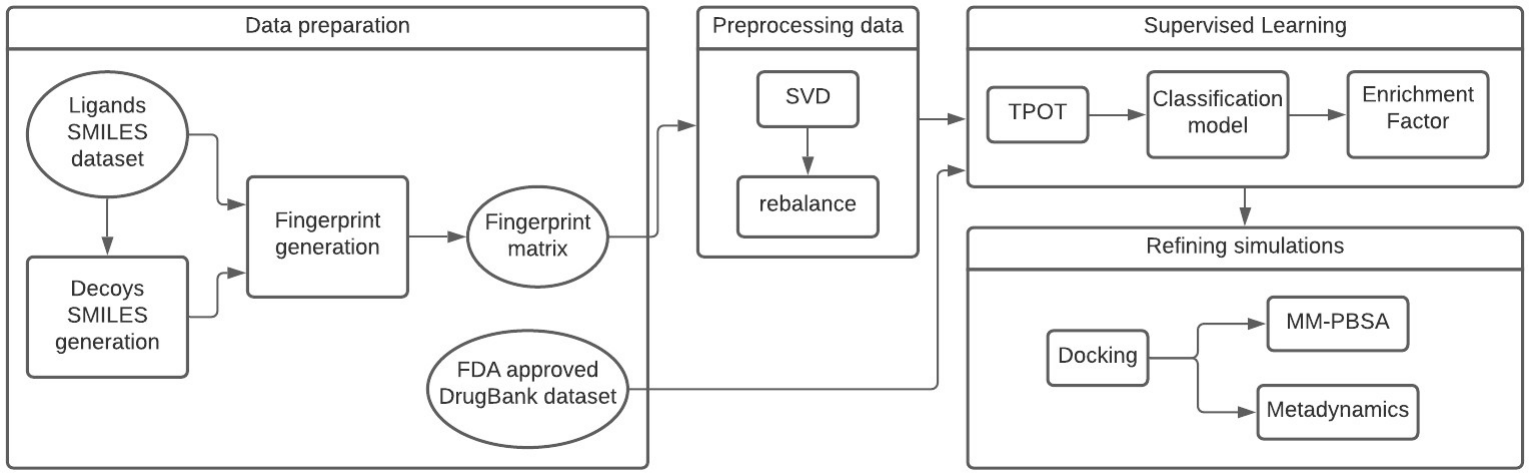
The workflow of our strategy. It is composed of four blocks: Data preparation; Preprocessing data; Supervised learning; and Refining simulations. Rectangles indicate processing steps and ellipsoids denote datasets.

This section details our computational strategy to predict candidate inhibitors for SARS-COV-2 M^pro^. (Fig 1) presents the workflow that outlines the process. We explain data collection and preprocessing, prediction of molecules through the in-house developed supervised learning strategy, and refinement of these molecules through docking and molecular dynamics.

### Data preparation

Our strategy intends to predict small molecules to favorable interact with a protein in an active pocket. As a first step, it is necessary to collect robust data to feed the machine learning strategy.

Only positive samples are insufficient to build a robust predictive model and, to make a model that correctly filters non-active compounds, is important to have inactive molecules in the dataset. One challenge in molecule screening modeling is how to label compounds as inactive since the range of inactive molecules is very wide, so we use decoys to fulfill this task. We generated 50 molecular decoys for each active ligand using the “Database of Useful Decoys: Enhanced” server (DUD-E) [33].

An important step in machine learning is feature engineering, where the raw data is converted into a more informative value, called a feature. In the drug repurposing context, we aim to convert the chemical data into an informative numerical feature understandable for predictive models. This is achieved by computational processing for molecular fingerprint generation, which are numerical features extracted from chemical structures that can be used to assess the similarity of two compounds.

The structural similarity of two molecules is commonly evaluated by computing the Tanimoto coefficient on their chemical fingerprints [26]. High values indicate two compounds are similar but do not provide information about specific chemical groups they share. This is because the compounds are compared one by one, resulting in a local point of view, unlike when compounds are analyzed together, achieving a global view. In this scenario, we decide to use techniques to increase the overall quality of the data and to help the machine learning strategy better capture the relationships between features, beyond the Tanimoto coefficient.

The active ligands and the decoys composes our dataset and we described the molecule features through a combination of two molecular fingerprints, implemented by RDKit [34]. The first of them is a topological-based fingerprint inspired by Daylight fingerprint that extracts chemical patterns from the chemical graph, and the other one is a structure keys-based fingerprint implementation of the public MACCS keys, indicating the presence or absence in the compounds of particular substructures or features from a given list of structural keys [26]. Thus, we built a data matrix containing numerical information about active molecules and decoys on the rows and fingerprint features on the columns. The resulting matrix is composed of 2214 columns and used as input to build the supervised machine learning model.

### Preprocessing data

The next step concerns processing the data in order to improve the predictive performance. Usually, the matrix data generated by fingerprints is sparse, because molecules have different sizes and functional groups. This results in many cells filled with zeros. Moreover, the assumption that similar molecules share similar activities might not always be valid, since minor modification on functional groups causes an abrupt change in activity [35]. Small changes of descriptor values can lead to considerable changes in molecular properties. Thus, before using raw fingerprints as input for a machine learning strategy, we perform singular value decomposition (SVD) that allows us to obtain lower-dimensional projections of the data [36]. The SVD reduces the dimensionality while preserving essential properties of the raw data matrix, detecting meaningful patterns while discarding weak signals and noises [37].

As 50 decoys were generated for each active molecule, there is an imbalance between actives and decoys, that is, positive and negative classes. Imbalanced data refers to a dataset in which one or more classes have much grater number of examples than others [38]. In drug discovery, the prevalent class, called majority class, comprises inactive molecules, while the rarest class, or minority class, is composed by active compounds. Generally, imbalanced data significantly challenges traditional machine learning models. To address this issue, a rebalance is performed before building the learning model, aiming to attain a more balanced input data and improve the model prediction capacity. We use a resampling technique to rebalance the sample space in order to relieve the effect of the skewed class distribution in the learning process. Given an imbalanced dataset, we split the majority class set into subsets similar in size to positive class. We combine each negative subset with the positive one, and create many smaller balanced subdatasets that will be used as input to an ensemble of classifiers, similar to the strategy implemented in [39].

### Supervised learning

The matrix of active compounds and their respective decoys for each target after preprocessing data served as training data to build a prediction model. To select an appropriated learning model and optimize its parameters for each dataset, we used TPOT [40], an automated machine learning system written in Python. The pipelines were trained and evaluated under 5-fold cross validation using scikit-learn library in Python [41].

Nearly 2,000 approved molecules were obtained from Drugbank [42] and screened against each target using the best pipeline according TPOT. This set of approved drugs was used as a validation set, aiming to verify if the best machine learning pipeline is able to retrieve active compounds from Drugbank FDA approved drugs. After screening, Drugbank approved compounds were ranked according to the class probabilities returned by the respective pipeline. Best ranked compounds can be selected as candidates for future analysis in more robust experiments, such as molecular docking and molecular dynamics. The metric used to assess the performance of the VS strategy is the Enrichment Factor (EF), which is a measure of how much the compound dataset is enriched with actives after applying the VS strategy [6]. Usually, a sample of one percent (top-1%) of the original data, after labeling probabilities, containing the best classified compounds is used to calculate the EF. This metric is computed as the ratio between the proportion of actives after and before applying the VS strategy.

$$\begin{array}{c} EF=\frac{\frac{a2}{a2+d2}}{\frac{a1}{a1+d1}} \end{array}$$ (1)

in which a1 = actives before the VS step, a2 = actives after the VS step, d1 = decoys before the VS step, and d2 = decoys after the VS step.

For our search for potential SARS-COV-2 M^pro^ inhibitors, we collected a benchmark on April 24, 2020 in PDB [43] consisting of 74 SARS-COV-2 main protease structures in complex with diverse ligands. The best machine learning algorithm calculated by TPOT under a 5-fold cross validation was the MLP Classifier, with parameters: alpha = 0.0001, learning_rate_init = 0.1, max_iter = 500, hidden_layer_sizes = (100,75,50,25,).

### Docking

After using our VS strategy on Drugbank FDA approved drugs, we selected 17 compounds candidates to interact against SARS-COV-2 M^pro^, excluding those that act on the central nervous system or are illegal—allowed to medical use under specific conditions. We executed the local docking focusing on the binding site: H41, S46, M49, Y54, F140, L141, N142, G143, C145, H164, M165, E166, L167, P168, H172, A173, F185, D187, Q189, T190, A191, and Q192 [10]. The grid box was elaborated using Autogrid from AutoDock Tool [44]. We added polar hydrogen to the macromolecule, intended to correct any deprotonations of the structure, and rotation was allowed to all rotatable bonds of the ligands. Every molecule was saved in pdbqt format so they could be used as input for AutoDock Vina [45]. We executed three simulations replica and we set the tool to generate seven poses for each drug in each replica. The best scored complexes were analyzed by Biovia Discovery Studio 2020 [46] to describe the contacts profile.

Docking indicates preferable areas where the ligands interact with the protein. However, this is a static result that can be improved with molecular dynamics approximating the system to real conditions. Moreover, the docking score function does not reflect the the binding energy, which can be estimated by molecular dynamics methods such as MM-PBSA and metadynamics.

### MM-PBSA simulations

We selected the six best-scored poses from docking for molecular dynamics simulations, MM-PBSA and metadynamic, using two distinct force fields: CHARMM [47] and AMBER [48]. These simulations intended to predict the binding energy for each complex and inspect the impact of the force field on this kind of simulation. Using two different ways to estimate the interaction power of the two molecules allows us to view the complex binding from more than one perspective. The use of distinct force fields allows us to see if even with different parameters governing the molecular trajectories, the tendency of the interaction force is maintained.

We performed the MM-PBSA on GROMACS 5.1.4 [49]. We followed the SwissParam [50] protocol to generate drugs topologies with the CHARMM force field [47] and antechamber [51] to generate drugs topologies with the AMBER force field [48]. Each system was centered on a dodecahedral box with a distance of 14 Å between the complex and the edges, solvated with TIP3P water, and neutralized with 0.15 mol.L^-1^ sodium chloride.

For energy minimization, we applied the steepest descent [52] and conjugate gradient [53] algorithms with convergence criteria of 0.24 kcal.mol^-1^ in both cases. Subsequently, we equilibrated the system in 300 K and 1.0 atm using seven simulations stages with restriction forces being gradually removed. The first stage was in canonical coupling and, the others, in isotherm-isobaric coupling. The simulation protocol consisted of the Verlet Leap-frog integration algorithm [54], with an integration step of 2.0 fs. Also, v-rescale algorithms [55, 56] with a *tau*_*t*_ = 0.1 ps and Parrinello-Rahman [57] with a *tau*_*p*_ = 2.0 ps were used for the temperature and pressure couplings, respectively. For the CHARMM force field, we applied the settings suggested by GROMACS for van der Waals parameters on the molecular dynamics parameters file.

Next, we submitted the complexes to 100 ns of molecular dynamics each, under the same parameters. From the trajectories obtained, we compressed it into 500 snapshots due to MM-PBSA calculation using g_mmpbsa tool [58] and default parameters.

Since the sample data set is small to compare the energies calculated with both force fields, only six points, we used Spearman’s rank correlation to assess the concordance of the results.

### Metadynamics

Metadynamics is an appropriate method to determine the binding energy for protein-ligand complexes, mainly when the active site is large and the ligands very small [59, 60] and it is a quicker simulation than MM-PBSA. This simulation also allows us to establish different sets of collective variables, which govern the simulation in specific ways. The combination of several of these sets of collective variables describes more clearly the unbinding behavior of the ligand from the active site of the protein.

We used the same six complexes to perform metadynamics simulations with the same force fields used previously, CHARMM, and AMBER. First, each system was solvated using the Solvate v1.7 plugin for Visual Molecular Dynamics software [61] with TIP3P water in a cubic box with 14 Å padding. Next, we neutralized the systems with 0.15 mol.L^-1^ sodium chloride using Autoionize v1.7 plugin for VMD software [61].

We used the NAMD 2.14 software [62] to perform the simulations with a time-step of 2.0 fs, in an NPT ensemble with Langevin thermostat and barostat devices set at 300K and 1 atm. We set periodic boundary conditions and a cutoff of 12 Å for the nonbonded interactions, and we calculated the long-range electrostatic interactions by the Particle mesh Ewald (PME) method. The systems were minimized for 1000 steps by minimization with conjugate gradient, then a protocol of relaxation with harmonic restrains was performed. This protocol consists of:

1. 500 ps MD with harmonic restrains in the M^pro^ and the ligand;
2. 500 ps MD with harmonic restrains in the backbone of the M^pro^ and ligand;
3. 500 ps MD with harmonic restrains in the M^pro^;
4. 500 ps MD without harmonic restrains;
5. 8 ns MD without harmonic restrains, restarting the velocities;
6. 500 ps MD with harmonic restrains in the backbone of the M^pro^;
7. 500 ps MD with harmonic restrains in the M^pro^;
8. 1 ns MD with harmonic restrains in the M^pro^, restarting the velocities.

For the actual metadynamics, we restrained the M^pro^ harmonically, and we kept the ligands free for all the systems during the 7 ns. We executed three simulation replica for each ligand with each force field. We set the height of the Gaussians to 0.02 kcal.mol^-1^, added every 1.0 ps with a width of 1.77. We chose two groups of collective variables (CV):

- **Group 1**: The first CV were the distance between the center of mass of M^pro^ C145 and the center of mass of the closest atoms of the ligand to M^pro^ C145, varying between 0 Å and 30 Å with an amplitude fluctuation of 2 Å. For the second CV, we established the angle between the center of mass of M^pro^ C145, the center of mass of the closest atoms of the ligand to M^pro^ C145 and the center of mass of the entire ligand, varying from 0º to 180º with an amplitude fluctuation of 10º.
- **Group 2**: The first CV were the same distance for group 1. For the second CV, we established specific angles for each ligand, considering their internal coordinate variations, varying from 0º to 180º with an amplitude fluctuation of 10º. The atoms involved in each angle are described in Table 1. The label of each ligand is available on Supporting Information.

**Table 1:** Set of atoms for each angle component for the selected ligands.

Ligand	Atoms involved in the CV		
	Set 1	Set 2	Set 3
Ambenonium (DB01122)	C145	O,O1,N1,N2,C8,C9,C10,C11	ligand
Plerixafor (DB06809)	C145	N,N1,N2,N3,C7,C8,C9,C10,C11,C12,C13,C14,C15,C16	N4,N5,N6,N7,C18,C19,C20,C21,C22,C23,C24,C25,C26,C27
Revefenacin (DB11855)	C145	H41,H04,H05,O3,N4,C31,C34	ligand
Mirabegron (DB08893)	H1,H2,H11,N1,N2,C8,C9,C10,S	ligand	H19,H20,H21,H22,H23,C15,C16,C17,C18,C19,C20
Diloxanide furoate (DB14638)	beta carbon of C145	O,O1,O2,C,C1,C2	O3,C4,C5,C6,C12
Vorinostat (DB02546)	C145	H1,H2,H14,H15,H16,H17,H18,H19,O1,O2,N1,C10,C11,C12,C13	ligand

We chose this pair of collective variables based on the principal movements of the ligands inside the protein on the equilibrium molecular dynamics stage. These CVs showed better sampling and convergence about the configurations on each minimum before the recrossing.

Despite operating the metadynamics by varying two collective variables, distance (*CV*_*dist*_), and a specific angle (*CV*_*ang*_), the unbinding process is mainly described by pull off the ligand from the protein. For this reason, we can estimate the metadynamics free energy onto the *CV*_*dist*_ according to the (Eq 2) [60].

$$\begin{array}{c} -\beta G\left(CV_{dist}\right)=ln\frac{\int e^{-\beta G(CV_{dist},CV_{ang})}dCV_{ang}}{\int e^{-\beta G(CV_{dist},CV_{ang})}dCV_{ang}dCV_{dist}} \end{array}$$ (2)

in which *β* = 1/k_b_T, where k_b_ is the Boltzmann constant 1.9858 x 10^-3^ kcal.mol^-1^·K^-1^, T = 300K, and G(*CV*_*dist*_, *CV*_*ang*_) is the free energy value for each distance and angle pair described in the PMF (Potentials of Mean Force) map.

Since Mpro is an enzyme with a shallow and exposed active site, the 7.0 ns of simulation maps conformations of the ligand onto the active site, the bulk, and even allows the molecule to interact with the protein again in other regions. Thus, it is critical to analyze the distance profiles and potential and electrostatic energies over time to select the most appropriate PMF files, since they are constructed by overlapping Gaussians during the simulation. These files describe the energies within the catalytic site and on the bulk to reconstruct the free energy landscape of the system before the ligand explores states beyond the two intended to avoid overestimating the energies in these two states. We calculated the distance in each frame using CPPTRAJ [63] and nonbonded energies using NamdEnergy. We analyzed the results with VMD software [61] and Python in-house scripts.

The binding free energy from each ligand with both force fields was determined by the subtraction of the minimum energy of the ligand inside the protein binding site (*G*^*in*^) and the minimum energy of the ligand in the water (*G*^*out*^), without the protein influence according to (Eq 3).

$$\begin{array}{c} \Delta G_{bind}=G_{CV_{ang}}^{in}\left(CV_{dist}\right)-G_{CV_{ang}}^{out}\left(CV_{dist}\right) \end{array}$$ (3)

This influence could be noticed from the distance profile, in which we established a cutoff of 20 Å to consider the ligand inside the protein, concomitantly with energy profiles. When the complex energy is null, it indicates no influence of the M^pro^ on the ligand.

For each ligand, we then performed six simulations with each force field. We calculated the final free energy by weighting the free energy of each simulation using the Boltzmann average, which highlights more favorable states, which are the more negative ones. The Boltzmann average applied for our systems can be written as the summation in (Eq 4). Thus with it was done for the MM-PBSA essay, we measured the concordance of the two force fields with Spearman rank correlation.

$$\begin{array}{c} \Delta G_{bind}=-kTln\left(\sum_{i=1}^{3}\sum_{j=1}^{2}\frac{e^{-G_{R_{i}CV_{j}}}}{kT}\right) \end{array}$$ (4)

in which *R*_*i*_ *CV*_*j*_ is the *i*^*th*^ replica with the *j* CV set.

## Results and discussion

This section discusses detailed results for each step of our method. First, we show that our supervised learning strategy is able to enrich compound datasets for three well known targets. Then our strategy is applied to point out potential molecules to inhibit SARS-COV-2 M^pro^ that will be refined through docking and molecular dynamics. Next, molecules that went through docking simulation and their respective scores are presented. Finally, the binding energies calculated through MM-PBSA and metadynamics simulations for two force fiels are shown.

### Supervised learning

To execute the methodology and validate it, we used the data of three targets that have a large amount of FDA approved drugs available: HIV-1 reverse transcriptase, 5-HT2A serotonin receptor, and H1 histamine receptor. We searched for specific ligands on BindingDB [30] and removed from the set all drugs deposited on DrugBank, used as method validation. Thus, we established a set of 1,492 ligands for HIV-1 reverse transcriptase (PDB 4B3P), 1,199 ligands for H1 histamine receptor (PDB 3RZE), and 1,974 ligands for 5-HT2A serotonin receptors (PDB 6A94). Based on these data, we followed the data preparation described and generated the matrix for the training set of the predictive model.

Table 2 shows the results achieved by our VS strategy. For each target, we used the TPOT to select the appropriated learning model and ran it on the Drugbank FDA approved data [64] to retrieve drugs already used in the treatment of diseases related to these targets. The results were satisfactory, highlighting the HIV-1 target, where an enrichment above a hundred was obtained, in other words, at least a hundred times better than random picking.

**Table 2:** Values of enrichment after apply the virtual screening strategy in three different datasets.

Target	Best Algorithm	Drugbank EF
HIV-1 reverse transcriptase	K-Neighbors Classifier	102.5
5-HT2A serotonin receptor	MLP Classifier	12.4
H1 histamine receptor	MLP Classifier	10.6

The enrichment factor (EF) measures how well the VS strategy filtered out positive ligands and is not directly related to the model used. For the HIV target assay, the model returned only three results above the established cutoff line, and for this reason, the EF was significantly high. The results for the other targets were not as good, but are still favorable since they indicate that the strategy selects better results for molecules that interact with the protein than for ligands not yet classified for this purpose. In this case, these other molecules that stood out in the model could be potential study targets to identify if they interact well with the protein.

Observing the results, the next step was modeling and screening compounds from Drugbank FDA approved drugs for the SARS-COV-2 M^pro^. Ligands in complex with the published X-ray crystal structure for this protein [10] can be used as examples in the training model, in order to find other molecules that share similar features, since that compounds with similar structures are expected to have similar biological activities [65]. To obtain these ligands we collected 74 SARS-COV-2 M^pro^ complexes from PDB [43] and executed our VS, which returned 28 ligands whose probability of being M^pro^ inhibitors is superior to 0.9. Among these, we selected to proceed to the docking step the 17 best-ranked ones, excluding those that act on the central nervous system or are illegal.

### Docking

The compounds selected for docking simulation are displayed in Table 3 with their respective vina and our LBVS scores.

**Table 3:** Top-scored ligands for SARS-COV-2 M^pro^ selected for docking calculation in Autodock vina.

Ligand	DrugBank ID	Vina score	LBVS score
Plerixafor	DB06809	-8.2	0.92
Revefenacin	DB11855	-7.6	0.90
Mirabegron	DB08893	-6.7	0.92
Ambenonium	DB01122	-6.1	0.96
Diloxanide furoate	DB14638	-5.9	0.94
Vorinostat	DB02546	-5.8	0.95
Acetarsol	DB13268	-5.4	0.90
Lacosamide	DB06218	-5.3	0.98
Procainamide	DB01035	-5.3	0.98
Alverine	DB01616	-5.0	0.91
Phenacemide	DB01121	-4.9	0.98
Acetaminophen	DB00316	-4.7	0.96
Isoniazid	DB00951	-4.6	0.91
Mephentermine	DB01365	-4.4	0.97
Levmetamfetamine	DB09571	-4.3	0.97
Phentermine	DB00191	-4.1	0.95
Pargyline	DB01626	-4.0	0.98

**Fig 2:**
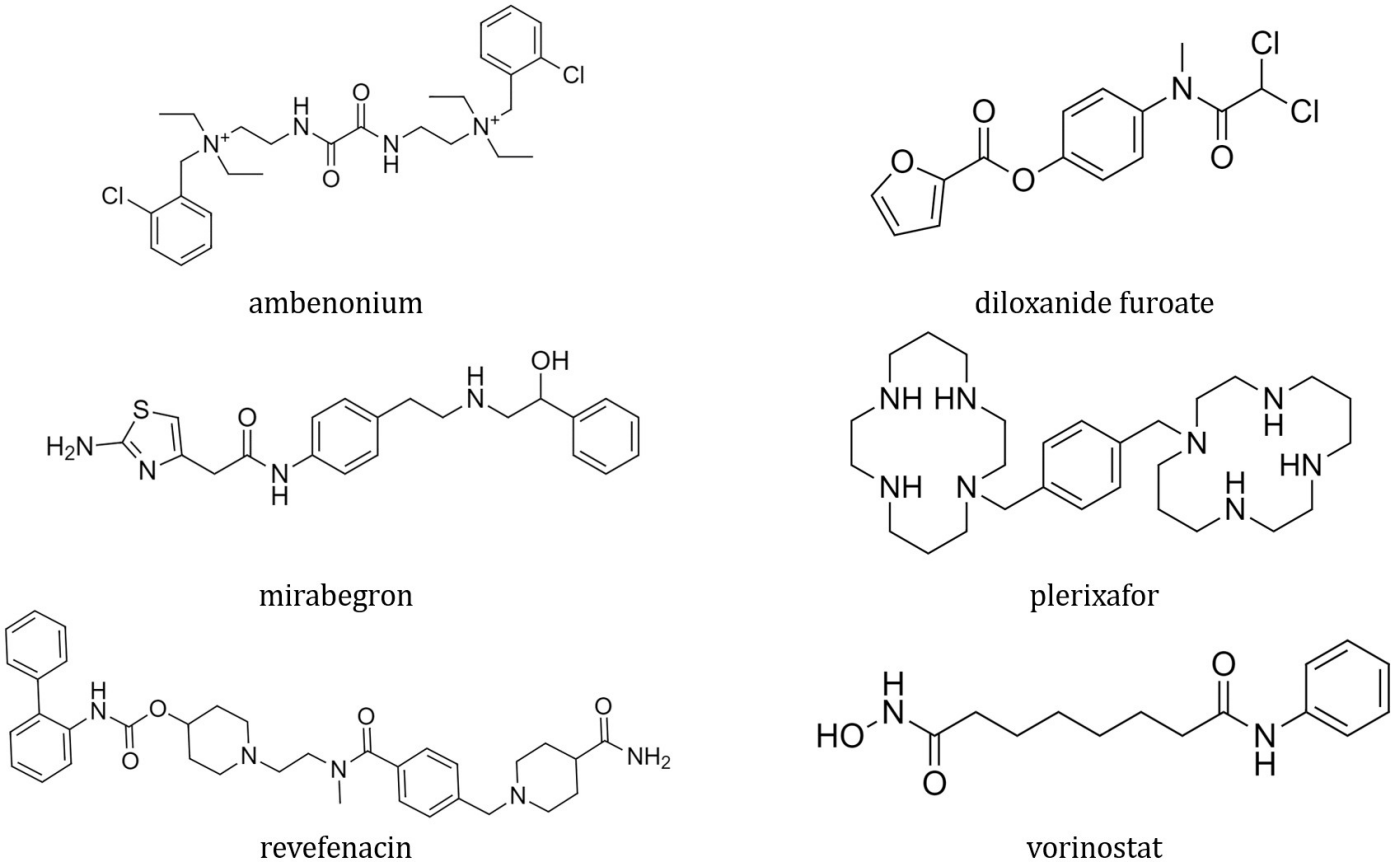
Compounds highlighted by docking assay. The 2D structure of the best scored ligands in docking experiment.

**Fig 3:**
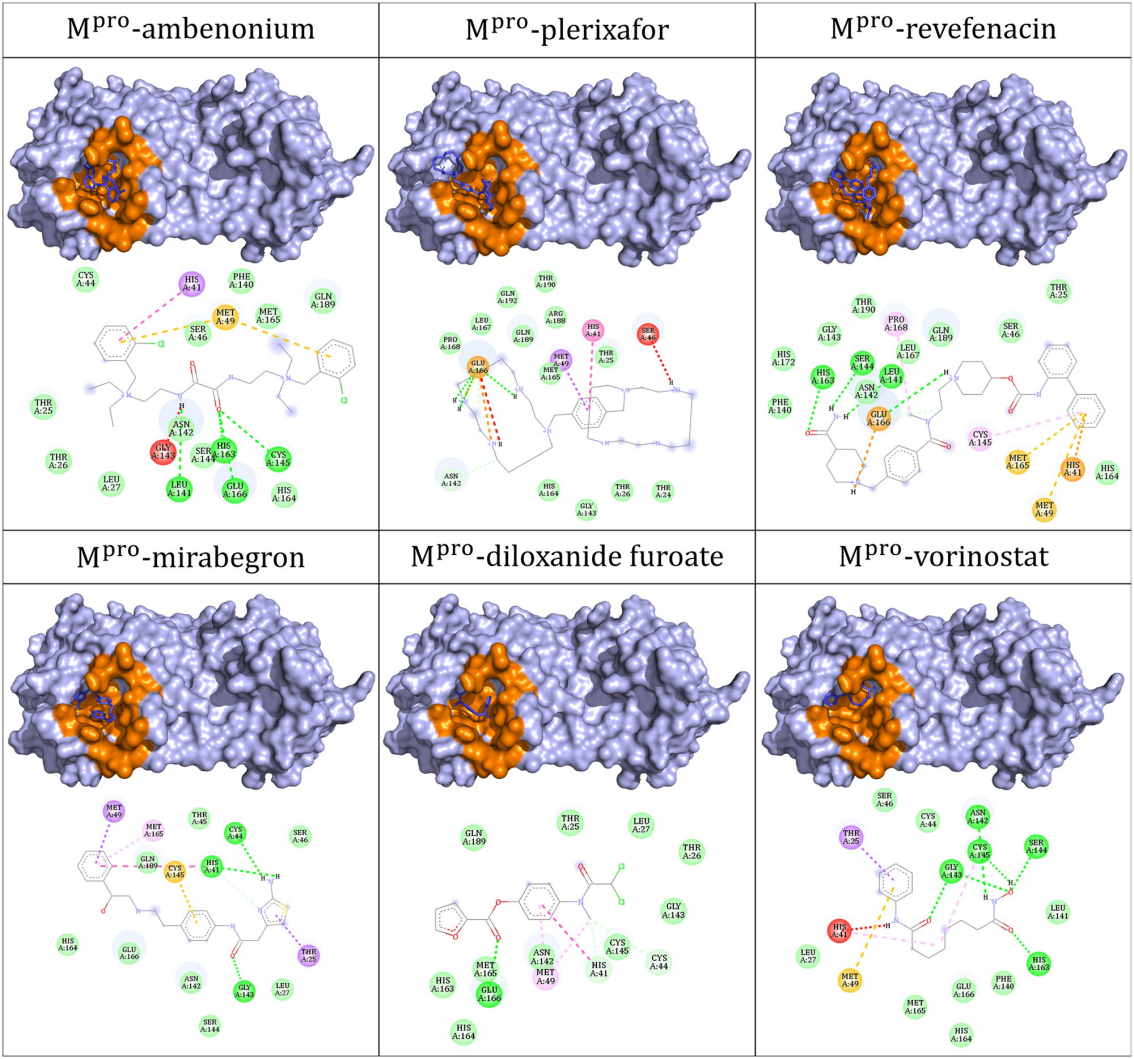
Top-scored complexes calculated by Autodock vina. The ligands (in blue) are well fitted in the M^pro^ active site (in orange). Hydrogen bonds are represented by green dashed lines; attractive electrostatic interactions, as orange dashed lines; orbital *π* interactions, as pink, yellow and purple dashed lines; unfavorable interactions, as red. Residues involved in hydrophobic interactions are represented by light green circles.

The poses with lowest vina scores show the preliminar favorable fit of the ligand inside the M^pro^ active site. The 2D structure of the six best ranked ligands through the docking assay are shown in (Fig 2). We can observe that there is diversity among the ligands and the interactions that they establish (Fig 3). However, some characteristics prevail, such as the aromatic character and the electronegative content due to nitrogen, oxygen, and chlorine atoms. This indicates the predominance of favorable interactions: hydrogen bonds, attractive electrostatic interactions, hydrophobic interactions, and some involving *π* orbitals. For M^pro^-vorinostat, there is only one unfavorable donor-donor interaction involving histidine 41. For M^pro^-ambenonium, there is one unfavorable electrical interaction between histidine 41 and one of the charged nitrogens. Besides, there is another unfavorable donor-donor involving glycine 143. For M^pro^-plerixafor there are two unfavorable donor-donor interactions, involving serine 46 and glutamic acid 166.

### MM-PBSA simulations

**Fig 4:**
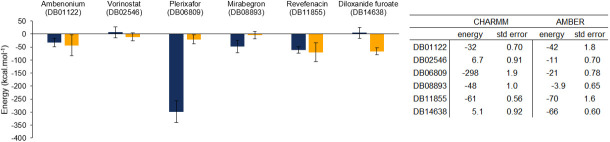
Binding energy on MM-PBSA calculation for the complexes. CHARMM is represented in blue and AMBER, in yellow. The error bars represents the standard error.

We chose the six vina best-scored poses to perform MM-PBSA (Molecular Mechanics Poisson-Boltzmann Surface Area) and (Fig 4) shows the binding energy calculated with the two distinct force fields.

The choice for a given force field impacts how the atoms interact with one another and how we calculate the dihedrals, which can lead the trajectories in distinct ways, even under the same conditions. This had a considerable influence on complexes’ behavior. The M^pro^-plerixafor complex was the one in which the energy contrasted the most considerably when varying the force field. This may have been due to its structure, which is composed of two wide loops, with nitrogens between carbons, attached to an aromatic ring. Because it is not an usual arrangement, the parameterization varied considerably with the force field and influenced the simulations in a wide range.

The other complexes also presented certain variations, but they were within the expected for MM-PBSA simulations, considering the peculiarities of each force field. It can be seen that the ligands ambenonium, vorinostat, and revefenacin were the least divergent because they are composed of predominantly open chains and without the presence of rarer groups. In the case of ligands mirabegron and dioxanide furoate, the influence on the parameterization that generated the energy difference may be due to the presence of an atom other than carbon in the middle of the aromatic ring, and each force field treats this differently.

The binding energies calculated with the MM-PBSA technique provided the estimated values with high oscillations, and it was very sensitive to the change of force field. We can see this from the Spearman rank correlation coefficient between the energies calculated based on both force fields, which is 0.143 with p-value 0.787. The detailed energy contributions are available on Supporting Information. Intending to have another perspective, we also calculated the binding energy with metadynamics using both force fields.

### Metadynamics

To calculate the binding energy, we choose among the PMF maps generated during each simulation the first one that described both the ligand inside and outside of the active site. This process is necessary because the simulation occurs with Gaussian potentials stacking according to the movements the complex assumes. Then, if we pick a PMF map in a simulation point much later than both events occurred, the energy may not be precise, because more Gaussian potentials will be considered [59].

**Fig 5:**
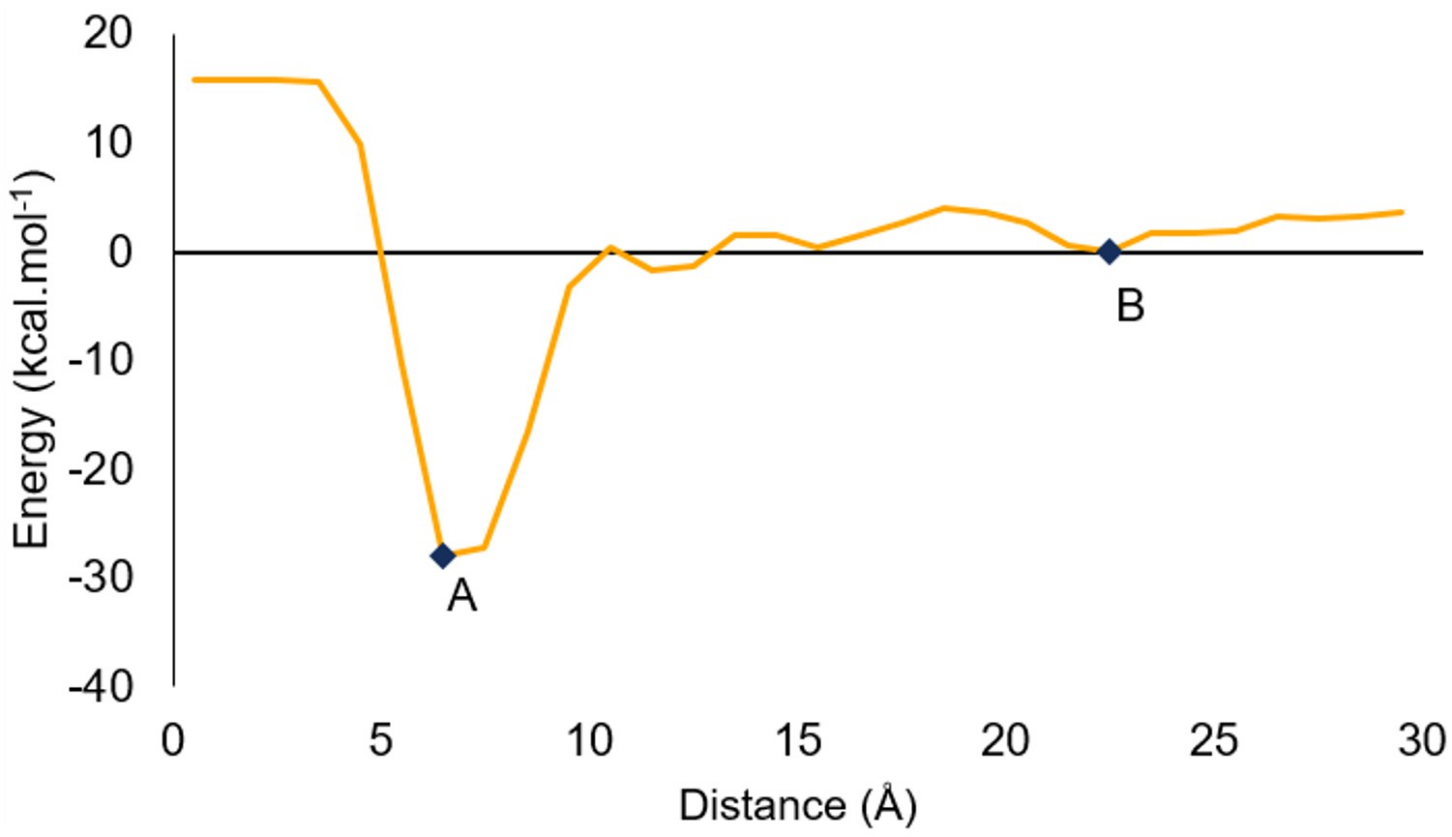
Energy profile for M^pro^-mirabegron in its first replica with CHARMM and the first CV set. The points A and B represents, respectively the minimum energy inside the active site and in the water.

Analyzing the distance and energy profiles together to calculate the binding free energy we can expect that the ligands did not have the same cutoff distance to indicate whether they were in the protein site or not, even though they were the same ligand but in different replicas. Considering the energies in this process makes the calculation more accurate, despite this cutoff variation. An example of these profiles of what was obtained for all simulations is available on (Fig 5). It represents the energy behavior along CV_dist_ for M^pro^-mirabegron. The other profiles are available in the Supporting Information.

Performing the calculation based on (Eq 4), we considered different modes of unbinding the ligand from the protein, some less energetic than others, but the influence of each is considered proportionally. It is important to emphasize that it is not possible to mix simulations executed with different force fields, because the parametrization influences translational and rotational movements of the molecules during the trajectory.

This possibility of different behavior according to the force field applied is the reason why the calculated energy, in each case, have a divergency, as already shown in MM-PBSA experiment. Table 4 explores the relation of the calculated energies with CHARMM and AMBER.

**Table 4:** Relation between calculated energies with CHARMM and AMBER. The energies described are in kcal.mol^-1^.

Ligand	CHARMM		AMBER	
	Energy	std error	Energy	std error
Ambenonium (DB01122)	-10	2.0	-17	3.0
Vorinostat (DB02546)	-15	1.2	-10	2.0
Plerixafor (DB06809)	-20	3.7	-20	3.4
Mirabegron (DB08893)	-35	8.7	-26	6.3
Revefenacin (DB11855)	-19	5.9	-17	4.4
Diloxanide furoate (DB14638)	-8.2	0.66	-13	1.6

Metadynamics had a more concordant behavior between the force fields compared to the MM-PBSA experiment. The Spearman rank correlation coefficient is 0.714 (p-value 0.111) when all points are considered. However, by removing the outlier which is the point corresponding to the complex M^pro^-vorinostat, this value reaches 0.9 (p-value 0.037) indicating that CHARMM and AMBER energy ranks are well correlated.

For CHARMM and AMBER, compound DB08893 (mirabegron) stands out from the others, and seems to be promising. Next, we notice DB06809 (plerixafor), which formed the lowest energy complex in MM-PBSA using CHARMM and the second-best ranked in the docking score. The other molecules do not follow a definite pattern for both force fields.

Another compound that we can highlight is DB01122 (ambenonium), a molecule that, to the best of our knowledge, had not yet been identified in any other study involving possible inhibitors for SARS-COV-2 M^pro^ in its active pocket. Although it was not the top-ranked compound in any of the docking and molecular dynamics simulations, it showed favorable interaction in all simulations. For these reasons, this ligand also ranks among the promising ones for further assays to prove inhibition activity.

Comparing all the steps performed, shown in Table 5, we can notice that the ligands end up changing their ranking during the virtual screening. The main difference occurred in the classifications of DB06809 (plerixafor) and DB11855 (revefenacin), which had the lowest LBVS scores of the set of the six best ligands but stood out in the other SBVS simulations. The opposite occurred with DB02546 (vorinostat) and DB14638 (diloxanide furoate). This variation demonstrates the relevance of combining ligand and structure based strategies for efficient inhibitor prediction, considering several positive aspects of both techniques.

**Table 5:** Ranking of compounds for each step of our method.

Supervised learning	Docking	MM-PBSA CHARMM	MM-PBSA AMBER	Metadynamic CHARMM	Metadynamic AMBER
DB01122	DB06809	DB06809	DB11855	DB08893	DB08893
DB02546	DB11855	DB11855	DB14638	DB06809	DB06809
DB14638	DB08893	DB08893	DB01122	DB11855	DB01122
DB08893	DB01122	DB01122	DB06809	DB02546	DB11855
DB06809	DB14638	DB14638	DB02546	DB01122	DB14638
DB11855	DB02546	DB02546	DB08893	DB14638	DB02546

## Conclusion

This work brings together an in-house developed machine learning strategy, docking, MM-PBSA calculations and metadynamics to identify FDA approved molecules that can potentially inhibit the main protease of SARS-COV-2. First, we devised a machine learning strategy that couples different molecule fingerprints to perform a first step of LBVS. Next, the resulting molecules go through SBVS steps, which consists of docking these molecules against the target protein (SARS-COV-2 M^pro^) using Autodock Vina and then selecting the poses with lowest vina scores. Finally, we selected the best-scored poses to perform MM-PBSA calculations and metadynamic simulations using CHARMM and AMBER force fields to predict the binding energy for each complex.

To assess the ability of our in-house developed machine learning strategy to retrieve potential candidates for the molecular docking and molecular dynamics, the Enrichment Factor was used, which is a measure of how much the compound dataset is enriched with active molecules after applying the screening strategy. Data for three well-studied targets, HIV-1 reverse transcriptase (PDB 4B3P), 5-HT2A serotonin receptor (PDB 6A94), and H1 histamine receptor (PDB 3RZE) went through screening using the proposed machine learning strategy and the EF was, respectively, 102.45, 12.4, 10.6, which are considered significant values. Regarding the identification of molecules approved by the FDA that can potentially act as inhibitors of the enzyme activity of the main protease of SARS-COV-2, this work points out six molecules, highlighting the strong interaction obtained for M^pro^-mirabegron complex. Among these six molecules, to the best of our knowledge, ambenonium has not yet been described in the literature as a candidate inhibitor for the SARS-COV-2 main protease in its active pocket.

The computational prediction of molecules that can potentially inhibit SARS-COV-2 main protease does not give the certainty that the top-ranked compounds inhibit the target, as it is an entirely computational experiment. Thus, as future work we envision to select the promising candidates for in vitro and in vivo studies that can show the inhibitory action of the proposed molecules for the target of interest.

## Supporting information

S1 Fig van der Waals energy on MM-PBSA calculation for the complexes. CHARMM is represented in blue and AMBER, in yellow. The error bars represent the standard error.
(TIF)

S2 Fig Electrostatic energy on MM-PBSA calculation for the complexes. CHARMM is represented in blue and AMBER, in yellow. The error bars represent the standard error.
(TIF)

S3 Fig Polar solvation energy on MM-PBSA calculation for the complexes. CHARMM is represented in blue and AMBER, in yellow. The error bars represent the standard error.
(TIF)

S4 Fig SASA energy on MM-PBSA calculation for the complexes. CHARMM is represented in blue and AMBER, in yellow. The error bars represent the standard error.
(TIF)

S5 Fig Labels for ambenonium atoms. (TIF)

S6 Fig Labels for plerixafor atoms. (TIF)

S7 Fig Labels for revefenacin atoms. (TIF)

S8 Fig Labels for mirabegron atoms. (TIF)

S9 Fig Labels for diloxanide furoate atoms. (TIF)

S10 Fig Labels for vorinostat atoms. (TIF)

S11 Fig Energy profile for M^pro^-ambenonium. Energy profile in all replicas (represented by the R1, R2 or R3) with AMBER (represented by A) and CHARMM (represented by C) and the two CV sets (represented by 1 or 2 preceding the force field label). The highlighted points represents, respectively the minimum energy inside the active site and in the water. The vertical axis represents the energy in kcal.mol^-1^ and the horizontal axis represents the distance in Å.
(TIF)

S12 Fig Energy profile for M^pro^-plerixafor. Energy profile in all replicas (represented by the R1, R2 or R3) with AMBER (represented by A) and CHARMM (represented by C) and the two CV sets (represented by 1 or 2 preceding the force field label). The highlighted points represents, respectively the minimum energy inside the active site and in the water. The vertical axis represents the energy in kcal.mol^-1^ and the horizontal axis represents the distance in Å.
(TIF)

S13 Fig Energy profile for M^pro^-revefenacin. Energy profile in all replicas (represented by the R1, R2 or R3) with AMBER (represented by A) and CHARMM (represented by C) and the two CV sets (represented by 1 or 2 preceding the force field label). The highlighted points represents, respectively the minimum energy inside the active site and in the water. The vertical axis represents the energy in kcal.mol^-1^ and the horizontal axis represents the distance in Å.
(TIF)

S14 Fig Energy profile for M^pro^-mirabegron. Energy profile in all replicas (represented by the R1, R2 or R3) with AMBER (represented by A) and CHARMM (represented by C) and the two CV sets (represented by 1 or 2 preceding the force field label). The highlighted points represents, respectively the minimum energy inside the active site and in the water. The vertical axis represents the energy in kcal.mol^-1^ and the horizontal axis represents the distance in Å.
(TIF)

S15 Fig Energy profile for M^pro^-diloxanide furoate. Energy profile in all replicas (represented by the R1, R2 or R3) with AMBER (represented by A) and CHARMM (represented by C) and the two CV sets (represented by 1 or 2 preceding the force field label). The highlighted points represents, respectively the minimum energy inside the active site and in the water. The vertical axis represents the energy in kcal.mol^-1^ and the horizontal axis represents the distance in Å.
(TIF)

S16 Fig Energy profile for M^pro^-vorinostat. Energy profile in all replicas (represented by the R1, R2 or R3) with AMBER (represented by A) and CHARMM (represented by C) and the two CV sets (represented by 1 or 2 preceding the force field label). The highlighted points represents, respectively the minimum energy inside the active site and in the water. The vertical axis represents the energy in kcal.mol^-1^ and the horizontal axis represents the distance in Å.
(TIF)
