# Supplementary figures and images for: Computational prediction of potential inhibitors for SARS-COV-2 main protease based on machine learning, docking, MM-PBSA calculations, and metadynamics
Source: PLoS One. 2022 Apr 22;17(4):e0267471. doi: 10.1371/journal.pone.0267471 (PMC9032443; doi:10.1371/journal.pone.0267471)

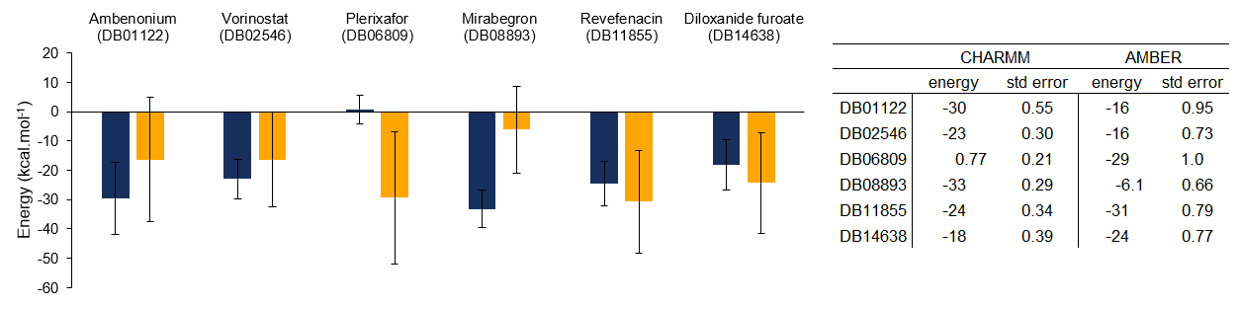

Supplement: S1 Fig — CHARMM is represented in blue and AMBER, in yellow. The error bars represent the standard error. (TIF) [file pone.0267471.s001.tif]

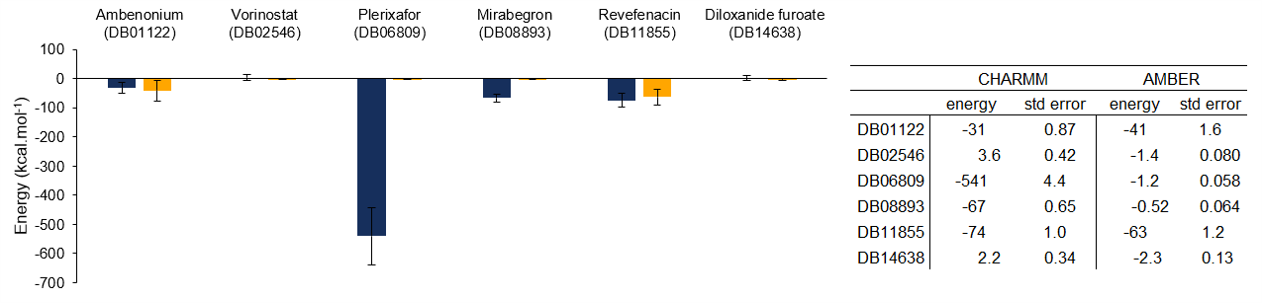

Supplement: S2 Fig — CHARMM is represented in blue and AMBER, in yellow. The error bars represent the standard error. (TIF) [file pone.0267471.s002.tif]

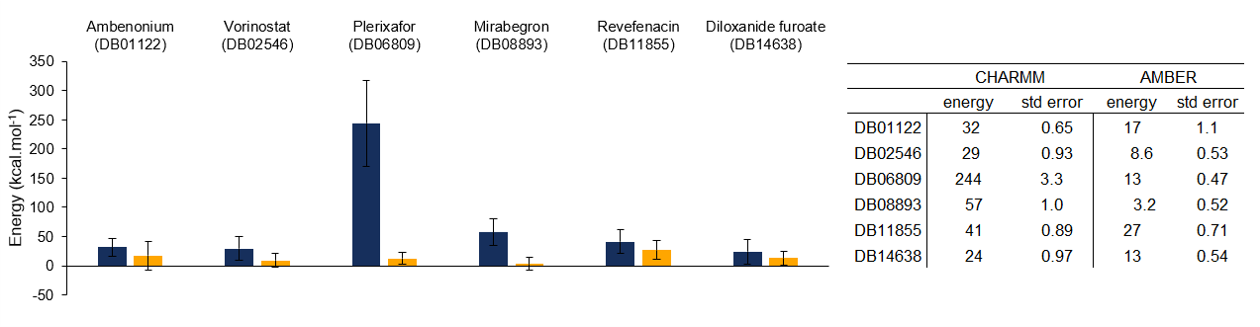

Supplement: S3 Fig — CHARMM is represented in blue and AMBER, in yellow. The error bars represent the standard error. (TIF) [file pone.0267471.s003.tif]

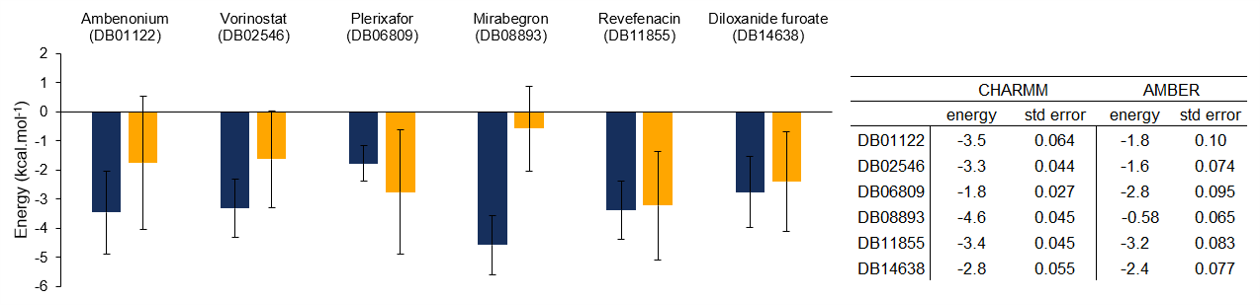

Supplement: S4 Fig — CHARMM is represented in blue and AMBER, in yellow. The error bars represent the standard error. (TIF) [file pone.0267471.s004.tif]

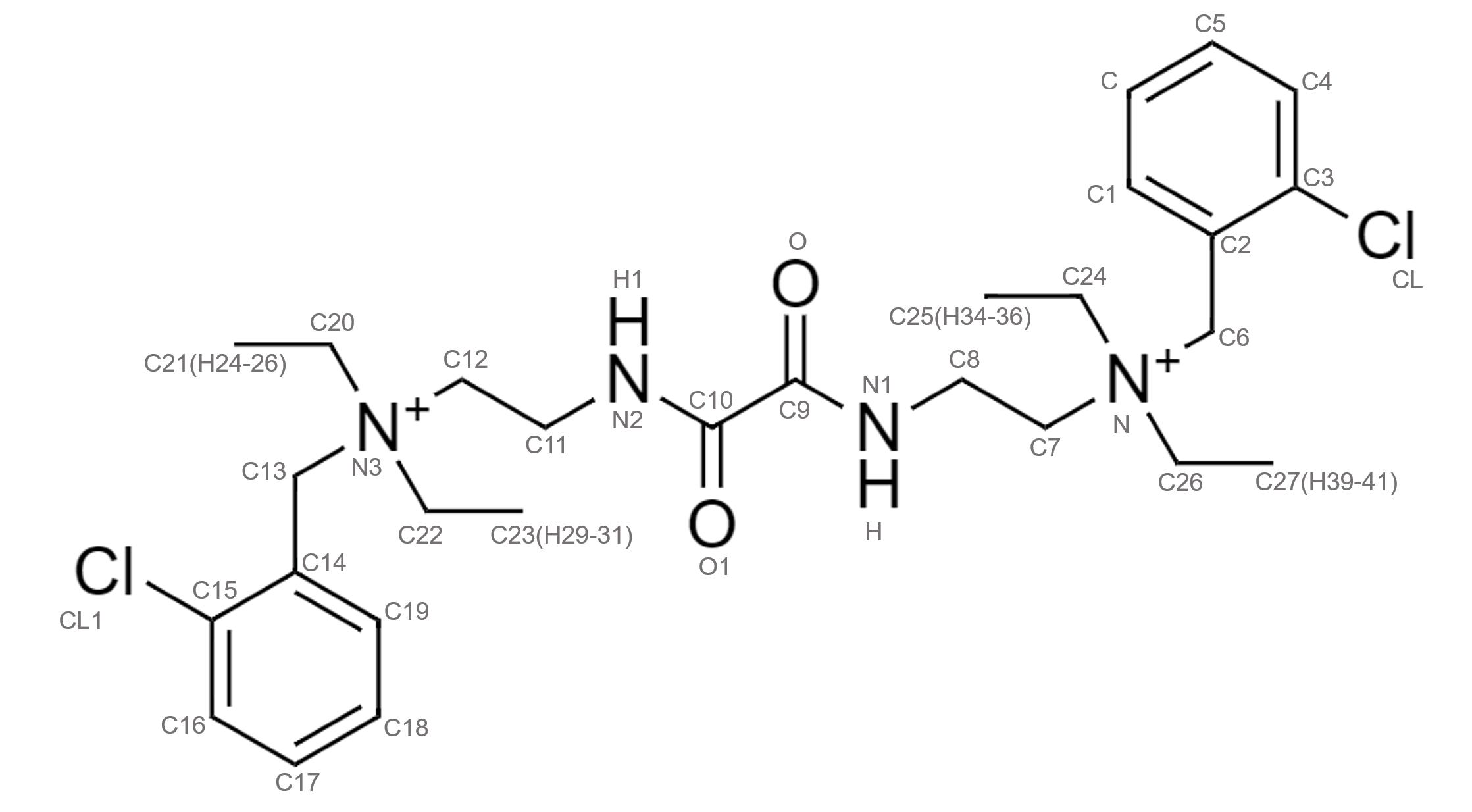

Supplement: S5 Fig — (TIF) [file pone.0267471.s005.tif]

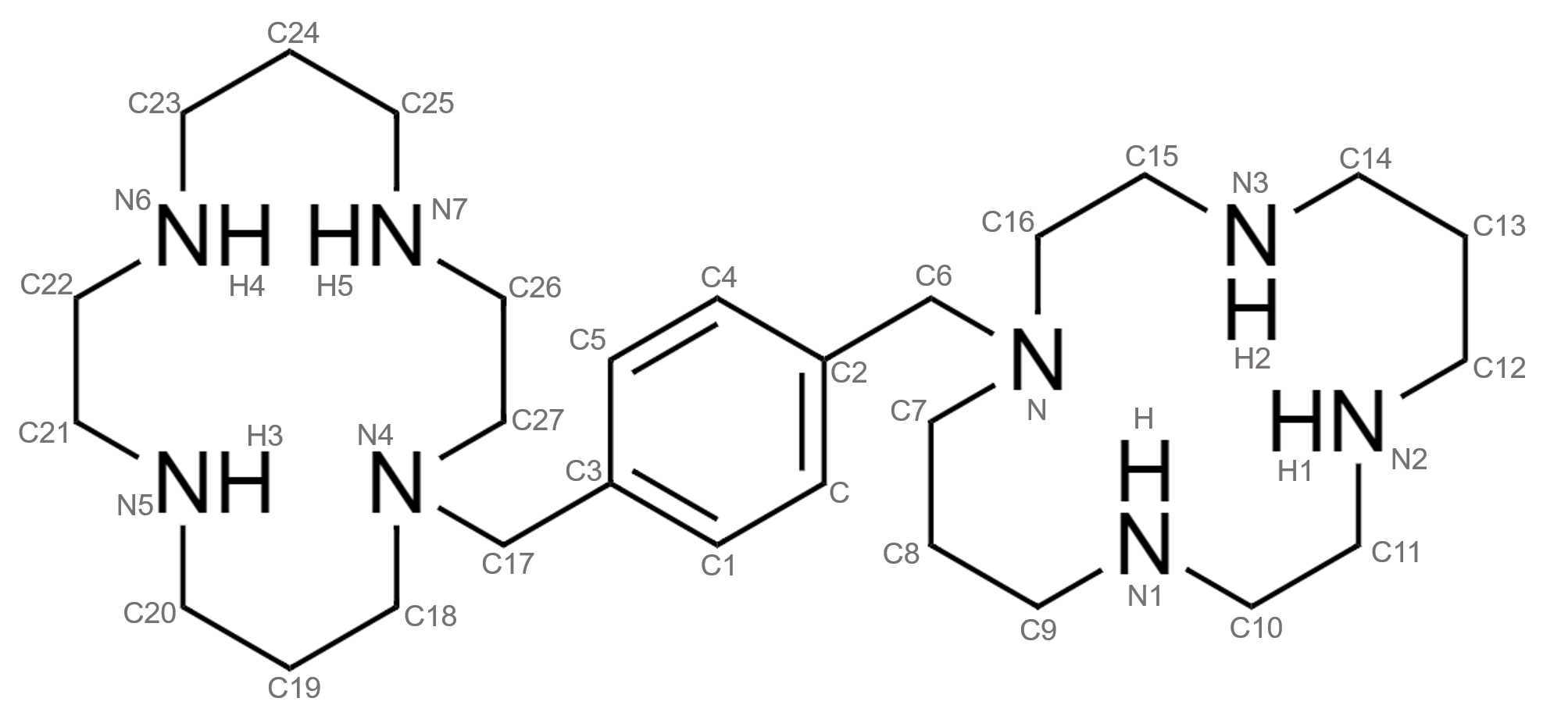

Supplement: S6 Fig — (TIF) [file pone.0267471.s006.tif]

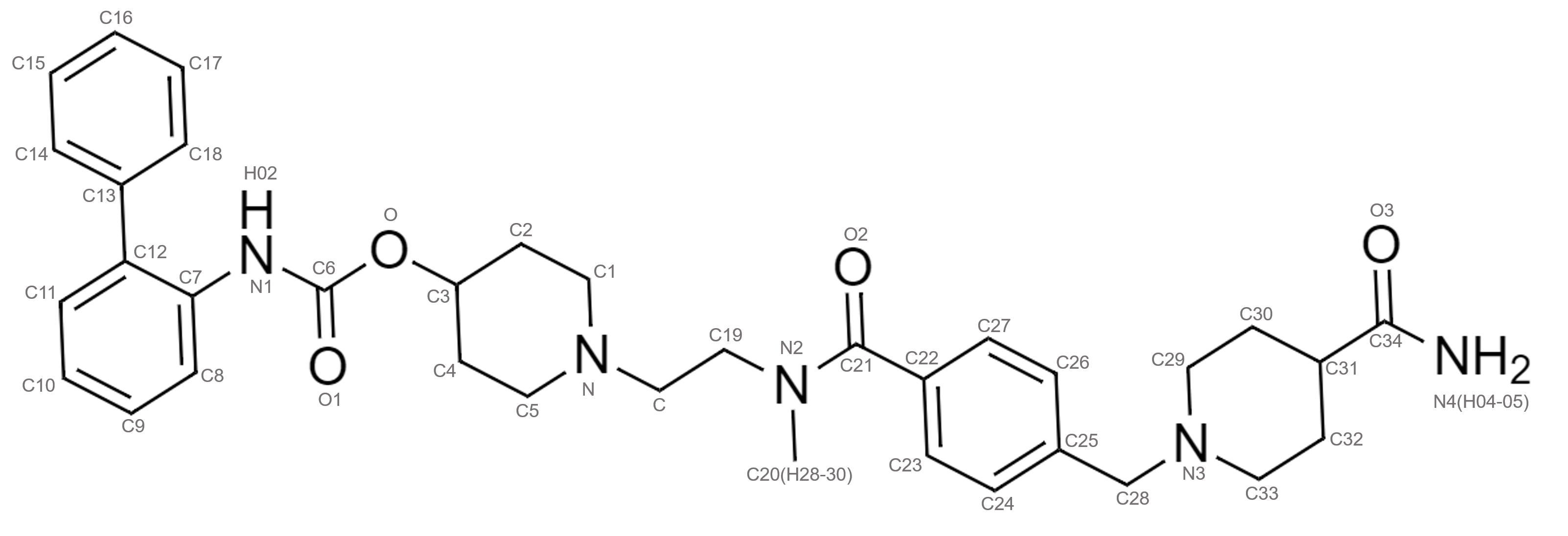

Supplement: S7 Fig — (TIF) [file pone.0267471.s007.tif]

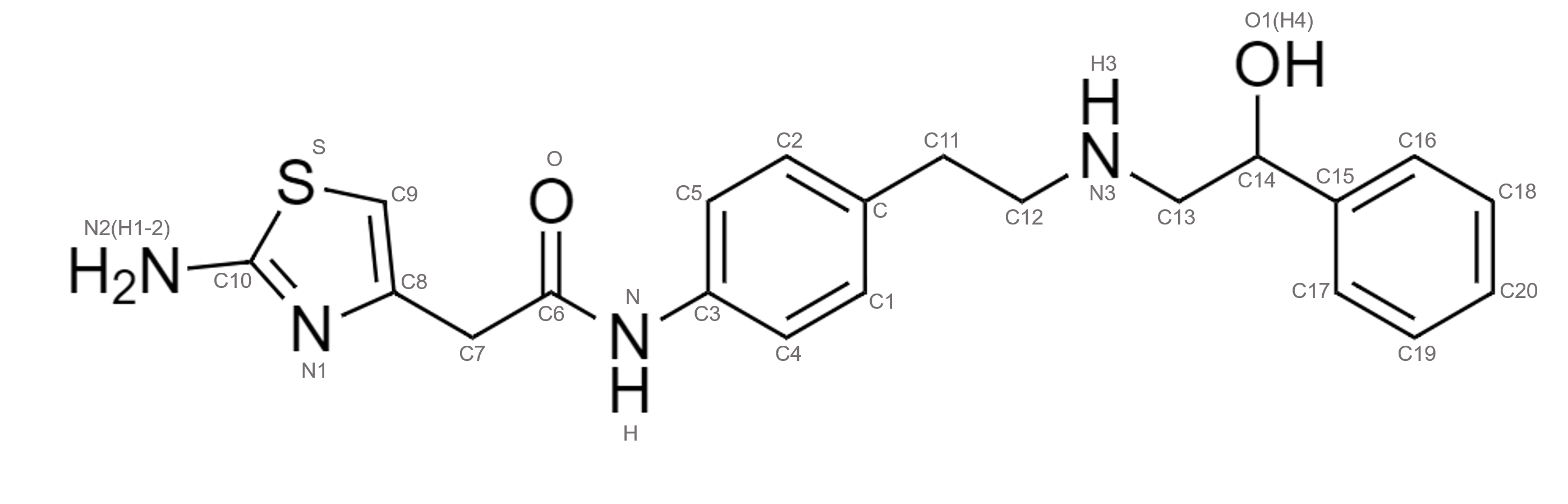

Supplement: S8 Fig — (TIF) [file pone.0267471.s008.tif]

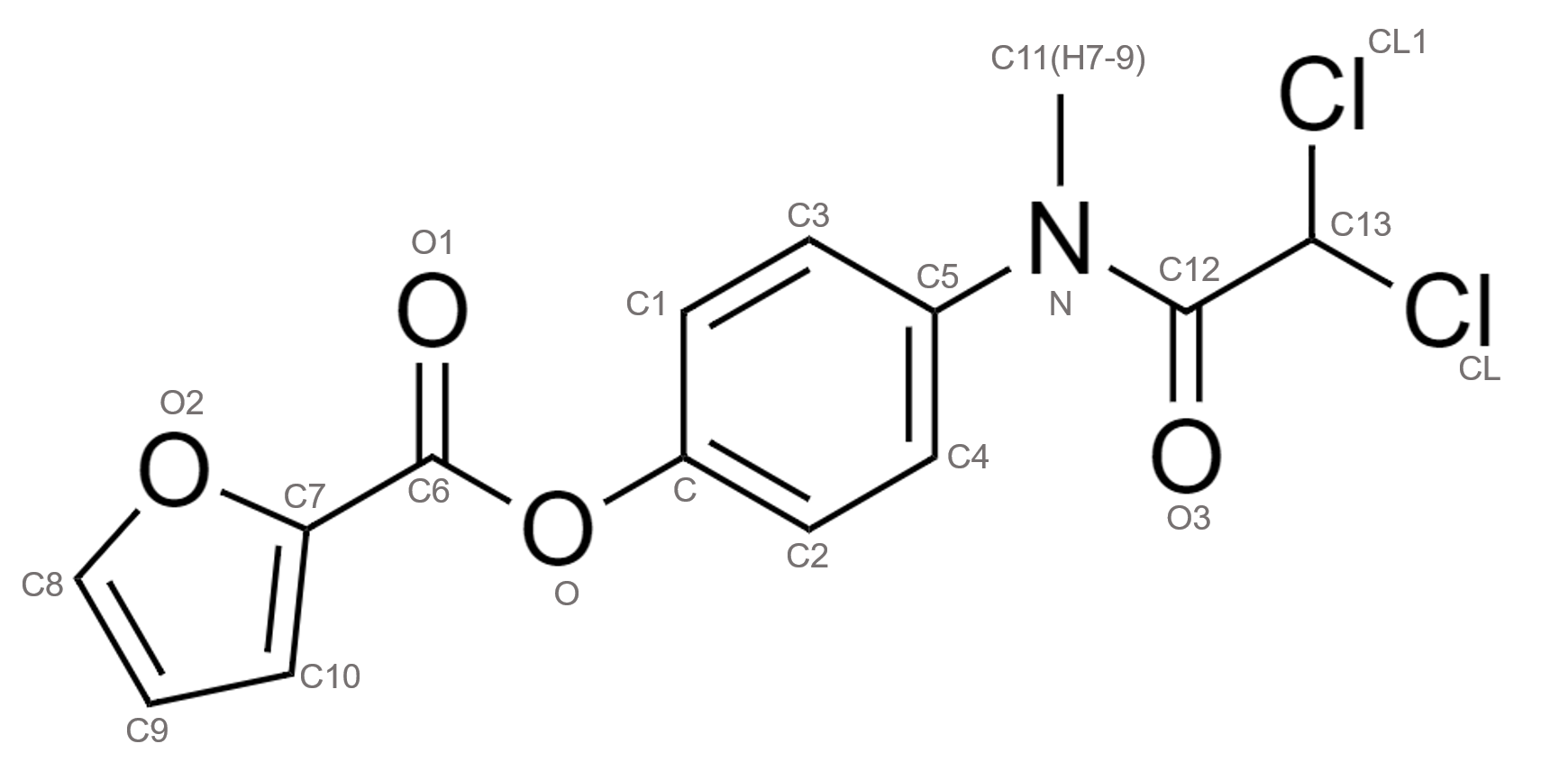

Supplement: S9 Fig — (TIF) [file pone.0267471.s009.tif]

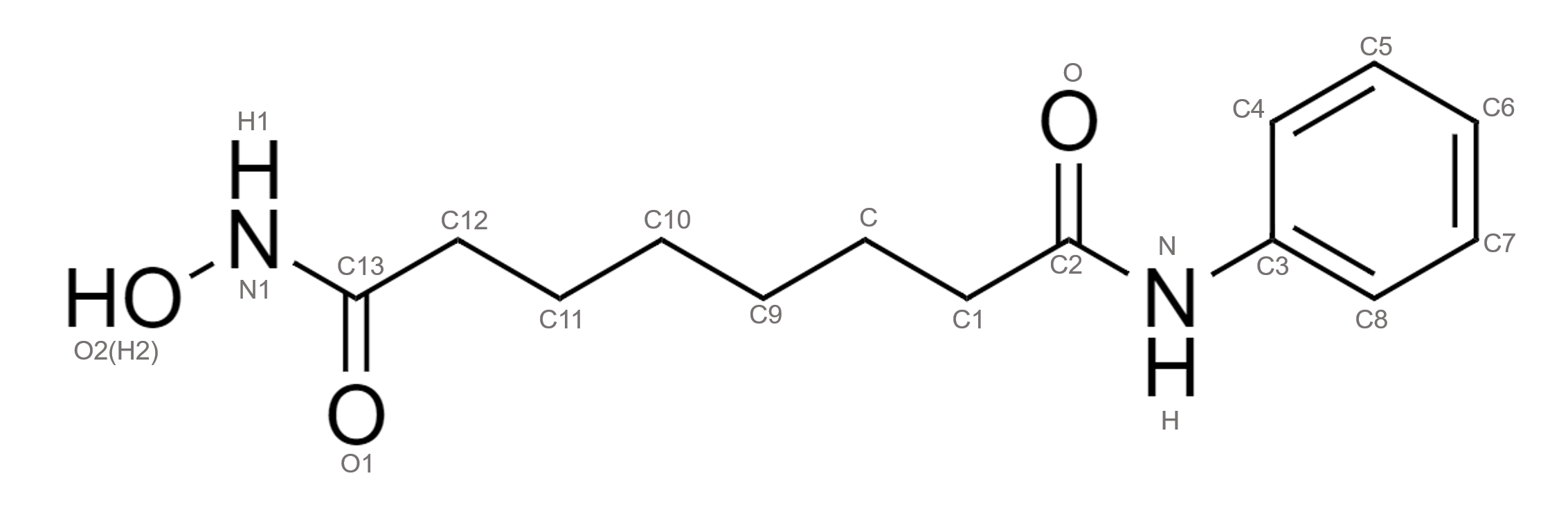

Supplement: S10 Fig — (TIF) [file pone.0267471.s010.tif]
